# Effect of emotional priming on eating willingness of women with restrictive diet

**DOI:** 10.3389/fpsyg.2024.1371484

**Published:** 2024-05-27

**Authors:** Yuchen Lv, Ying Chen, Weirui Xiong

**Affiliations:** School of Educational Science, Chongqing Normal University, Chongqing, China

**Keywords:** emotional priming, supra-threshold emotion, subliminal emotion, restrictive diet, eating willingness

## Abstract

**Background:**

There is controversy regarding whether negative or positive emotions have a greater impact on the eating behavior of restrictive dieters. Moreover, it is unclear whether unconscious emotional processing can predict the eating behavior of restrictive dieters. This study investigated the effects of conscious and unconscious emotional processing on the dietary behavior of women with restrictive diet.

**Methods:**

Female student participants (*N* = 600) completed the Dutch Eating Behavior Questionnaire to screen 60 each of successful, unsuccessful restrictive and unrestricted eaters. They were randomly and equally divided into two groups for supra-threshold and subliminal emotional priming and carried out a behavioral task to index eating intentions.

**Results:**

The restrictive dieters increase their willingness to eat when they are in a positive mood, even if they are not consciously aware of their happiness. Furthermore, the unsuccessful restrictive dieters are more intense.

**Conclusion:**

This study presents empirical evidence on the impact of positive emotions on the eating intention of restrictive dieters and the cognitive characteristics of unsuccessful restrictive dieters. Additionally, it offers guidance for unsuccessful restrictive dieters to personalize their treatment goals.

## 1 Introduction

Women have gradually become a high incidence group of restrictive eating behaviors in order to cater to the mainstream “thin is the beauty” in society (Leng et al., 2020). Restricted diet was first put forward by Herman and Mack (1975), which is a dietary behavior pattern aimed at controlling or restricting food intake to maintain or reduce weight. Actually, restrictive diet is generally regarded as a healthy and adaptive behavior, especially for overweight or obese people (Beames et al., 2016). However, extreme dieting behaviors can lead to potential problems, such as anorexia nervosa and bulimia (Madison and Mazzeo, 2022). Therefore, it is crucial to comprehend the dietary behavior and influencing factors of restrictive dieters.

According to Wang and Chen (2019), emotions can impact human eating behavior. However, it is still debated whether positive or negative emotions trigger increased eating in restrictive dieters. Cools et al. (1992) and Cavallo and Pinto (2001) found that positive and negative emotions can increase food intake among restrictive dieters, with similar effects. Xu et al. (2013) also verified the results. However, some studies suggest that only negative emotions lead to overeating in restrictive dieters (Erskine and Georgiou, 2010; Côté et al., 2016). Wei (2013) found that under negative emotions, restrictive dieters with high disinhibition tended to pay attention to food clues, and then their inhibition and control ability was also destroyed (Xi and Chen, 2017). In addition, Liu et al. (2020) pointed out that negative mood is a significant factor that hinders the success of dieting. In contrast, Yeomans and Coughlan (2009) found different results: positive emotions may have a greater impact on food intake among restrictive dieters than negative emotions. One possible reason for the inconsistent results may be that previous studies did not distinguish the types of restrictive dieters, while the influence of emotions on different restrictive dieters may be different. The restrictive dieters exist in two different groups. One is the successful dieters, which can achieve the goal of dieting. The other is the unsuccessful dieters, who always fail to diet. Research shows that successful dieters have stronger self-regulation mechanism than unsuccessful dieters, so that they can successfully resist the temptation of food and maintain the goal of dieting (Shah et al., 2002). Contrary to restrictive dieters, unrestricted dieters have no dieting goals. Furthermore, another possible reason is that the subjects have a defensive reaction to emotional materials in the experiment, thus masking their own psychological process (Redhead et al., 2016), which leads to the deviation between the experimental results and reality. Remarkably, Zhu et al. (2023) pointed out that adopting subliminal emotional priming paradigm is widely used to explore the nature of emotional problems.

In summary, on the basis of investigating whether there are differences in the effect of positive and negative emotions on the dietary behaviors of restrictive dieters, this study will (1) distinguish among various subcategories of restrictive dieters and investigate the circumstance surrounding their willingness to eat when they are influenced by both positive and negative emotions; (2) Assess the dietary behaviors of restrictive dieters under the conditions of both levels of emotional priming, namely supra-threshold emotional priming and subliminal emotional priming.

## 2 Study 1: effect of supra-threshold emotional priming on eating willingness of restrictive dieters

The experiment used the paradigm of supra-threshold emotional priming and adopted a two-factor mixed design of 2 (priming emotion: positive/negative emotion) × 3 (participant type: successful/unsuccessful/unrestricted dieter). In this design, the participant type serves as the between-subject variable, while the priming emotion functions as the within-subject variable. The dependent variable is the index of the average individual’s desire to eat. The experiment includes the task of priming positive emotions and the task of priming negative emotions. In the formal experiment, the presentation order of positive and negative tasks is balanced among the participants.

### 2.1 Methods

#### 2.1.1 Participants

Three hundred Dutch Eating Behavior Questionnaire (DEBQ) (Strien et al., 1986) were distributed at a university in Chongqing. Following the criteria outlined by Weng et al. (2012), we selected 30 successful dieters, 30 unsuccessful dieters, and 30 unrestricted dieters, all of whom were women (see Table 1). Due to the date of a successful restrictive dieter was incomplete, the dieter’s data was excluded. There was no significant difference in BMI or hunger degree among the three groups (*p* > 0.05).

**Table 1:** Demographic data of study 1 participants (M ± SD).

		BMI	Hunger degree	Restrictive score	Feeding score
Participant type	Unrestricted dieters	20.70 ± 2.79	3.13 ± 2.11	2.35 ± 0.45	2.66 ± 0.62
	Successful dieters	21.69 ± 3.11	2.86 ± 1.66	3.48 ± 0.40	2.57 ± 0.28
	Unsuccessful dieters	20.89 ± 3.02	3.13 ± 2.06	3.60 ± 0.41	3.41 ± 0.28
	F	0.91	0.19	79.77***	35.20***

*p < 0.05, **p < 0.01, ***p < 0.001.

#### 2.1.2 Materials

The DEBQ, developed by Strien et al., includes three sections: restricted dieting, emotional eating, and external eating. These assess conscious food restriction, eating in response to emotions, and the impact of external cues on eating habits, respectively. The questionnaire contains 33 items, scored on a 5-point scale. The internal consistency coefficients of the three sub-questionnaires are 0.95, 0.81, and 0.95, respectively. The questionnaire showed good reliability and validity in different studies in China (Shi et al., 2011; Kong, 2012; Weng et al., 2012). DEBQ can distinguish restricted dieters in more detail, which can be divided into unsuccessful dieters (restriction score ≥ 3 and eating score ≥ 3), successful restricted dieters (restriction score ≥ 3 and eating score < 3), and unrestricted dieters (restriction score < 3), where the restriction score is the average score of the restricted eating sub-questionnaire and the eating score is the average score of the emotional eating and external eating sub-questionnaires (Weng et al., 2012).

##### 2.1.2.1 Food pictures

We randomly selected 40 pictures from Kong (2012)'s library, comprising 20 high-calorie and 20 low-calorie food images. Selection criteria emphasized variety and visual appeal. To validate these images, experts in nutrition and psychology rated them to ensure accurate representation of calorie content and emotional impact. Adjust all the pictures to 450 × 337 pixels, and the background is black. Twenty-two female college students rated their delicacy, pleasure, and arousal on a 9-point scale. There is no significant difference between high and low-calorie pictures in three dimensions: palatability (*t* = 0.378, *p* > 0.05), pleasure (*t* = 0.646, *p* > 0.05), and arousal (*t* = 1.453, *p* > 0.05), which supports the effectiveness of food evaluation. Finally, 15 high-calorie and low-calorie food images were screened out.

##### 2.1.2.2 Emotional pictures

24 emotional pictures (12 happy pictures and 12 sad pictures) were randomly selected from the Chinese Affective Picture System (CAPS) in China (Bai et al., 2005). These pictures were processed and adjusted to 260 × 300 pixels with a black background. Twenty-two female college students rated all pictures for pleasure, arousal, and dominance using a 9-point scale. There is a significant difference in pleasure between positive and negative emotional pictures (*t* = 20.62, *p* < 0.001). However, there is no significant difference in arousal and dominance (*p* > 0.05), which indicates that arousal and dominance of emotional pictures are matched and balanced. Finally, according to the evaluation results, eight emotional pictures (positive/negative half) were selected as experimental materials.

#### 2.1.3 Procedure

**Figure 1:**
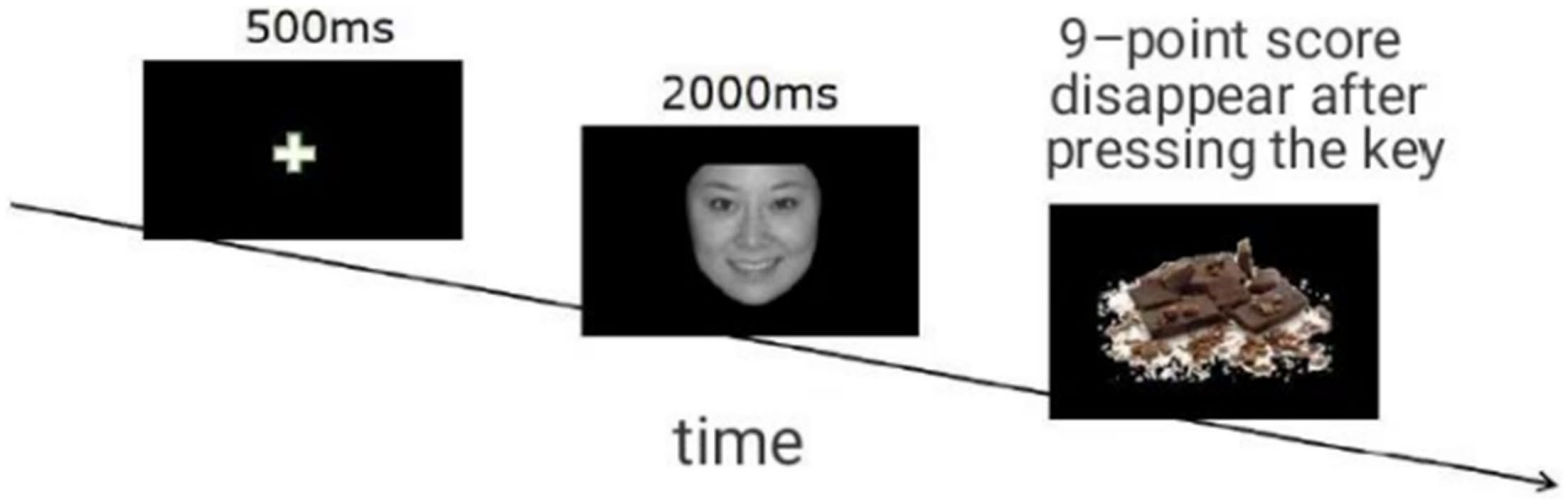
Flow diagram of study 1. Facial image reproduced with permission from Bai et al. (2005). Food image reproduced with permission from Kong (2012).

The experiment was conducted in a quiet laboratory, and the pictures were presented on a 24-inch display screen with a refresh rate of 60 Hz and a resolution of 1,024 × 768, and the participants were about 60 cm away from the screen. The experiments were all scheduled to start 20 min after the participants had a standardized light snack (or provide details if there were any specific dietary guidelines given to the participants). Before the experiment began, the participants were asked to rate their current hunger level (0 is not hungry, 10 is very hungry) and mood (1 is extremely unhappy, 9 is extremely happy). Then, the experiment (see Figure 1) was carried out. First, the participants were informed of the experimental tasks and entered the practice. After the practice, a formal experiment was carried out. The procedure began with a 500-ms gaze point “+” appearing in the center of the screen. This was followed by the random presentation of a startup picture for 1,000 ms, after which the food picture was shown. Participants need to rate their willingness to eat at 9 points, where “1” means they do not want to eat very much, and “9” means they want to eat very much. After pressing the key, the food screen disappears, and there is a time interval of 500 ms between the two trials the experiment was divided into two parts. Positive and negative emotional priming was carried out separately. Each part had 2 (priming map) × 30 (food map) combinations, and each combination was repeated once, for 120 trials. The two parts were separated by 15 min, and after each part, the participants were asked to evaluate their current emotions again to test the effect of emotional arousal.

### 2.2 Results

#### 2.2.1 Emotional priming effect test

The emotional score before the experiment was analyzed by variance with the emotional score after completing positive tasks and the emotional score after completing negative tasks. The results showed that *M_pre_* = 5.67, SD*_pre_* = 1.26; *M_positive_* = 6.45, SD*_positive_* = 1.21; *M_negative_* = 4.26, SD*_negative_* = 1.28; *F* (2, 87) = 126.96, *p* < 0.001. After conducting multiple LSD comparisons, there were statistical differences among the three emotional scores: The negative emotional score < the pre-test emotional score < and the positive emotional score. These results clearly indicate that the emotional manipulation achieved through the presentation of positive and negative pictures effectively alters participants’ emotional states, confirming the effectiveness of the emotional awakening operation, a technique used to elicit and measure emotional responses.

#### 2.2.2 Eating willingness of successful/unsuccessful/unrestricted dieters in the state of supra-threshold emotional priming

The analysis of variance, with emotional valence and participants’ grouping as independent variables and willingness to eat as the dependent variables, revealed a substantial effect of emotional valence (*F* (1, 86) = 75.12, *p* < 0.001, *η*2 = 0.466). Willingness to eat in positive emotions was significantly higher than that in negative emotions. The analysis reveals a significant interaction effect (*F* (1, 86) = 4.45, *p* = 0.015, *η*2 = 0.094), indicating that the interplay between two factors, likely “emotions” and “dieting status,” significantly impacts appetite.

Under the influence of positive emotions, our findings indicate that the appetite of unsuccessful dieters is significantly higher than that of both unrestricted dieters (*p* = 0.006) and successful dieters (*p* = 0.018), although there is no significant difference in the appetite of successful dieters and unrestricted dieters (*p* = 0.984). In contrast, when considering negative emotions, we observed that the appetite of unsuccessful dieters tends to be higher than that of successful restricted dieters (*p* = 0.063), though this difference does not reach statistical significance at the conventional threshold (*p* > 0.05). Furthermore, there was no significant difference found between the appetite of unrestricted dieters and successful dieters (*p* = 0.789), nor between successful restricted dieters and non-restricted dieters (*p* = 0.351), as indicated in Table 2.

**Table 2:** Eating willingness of three groups of participants in positive and negative supra-threshold emotional arousal state (M ± SD).

	Unrestricted dieters (*n* = 30)	Successful dieters (*n* = 29)	Unsuccessful dieters (*n* = 30)
Positive	5.10 ± 1.07	5.21 ± 1.32	6.12 ± 1.33
Negativity	4.59 ± 1.10	4.15 ± 1.27	4.83 ± 0.91

## 3 Study 2: influence of subliminal emotional priming on eating willingness of restrictive dieters

The experimental design replicates the participant selection, materials, and procedures of Study 1, with the main difference being the use of a subliminal emotional priming paradigm.

### 3.1 Methods

#### 3.1.1 Participants

The selection criteria mirror the first study. Out of 300 female college students, 30 are categorized as successful dieters, 30 as unsuccessful dieters, and 30 as unrestricted dieters (see Table 3).

**Table 3:** Demographic data of study 2 participants (M ± SD).

		BMI	Hunger degree	Restrictive score	Feeding score
Participant type	Unrestricted dieters	20.21 ± 2.83	2.67 ± 2.00	2.25 ± 0.46	2.69 ± 0.55
	Successful dieters	21.43 ± 2.40	3.23 ± 1.74	3.42 ± 0.31	2.51 ± 0.34
	Unsuccessful dieters	20.53 ± 2.85	3.40 ± 1.79	3.72 ± 0.47	3.32 ± 0.30
	F	1.64	1.30	102.59***	32.32***

*p < 0.05, **p < 0.01, ***p < 0.001.

#### 3.1.2 Materials

The experimental materials are the same as those in study 1.

#### 3.1.3 Procedure

**Figure 2:**
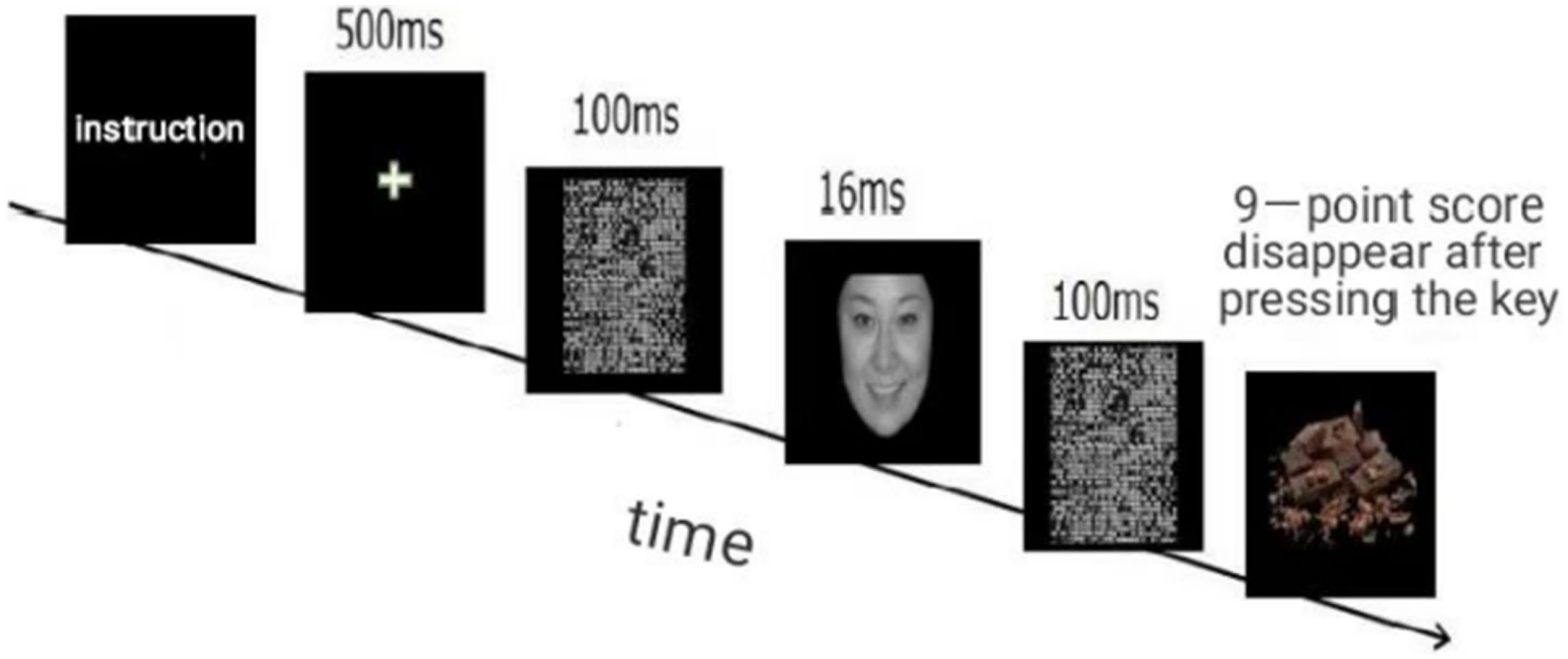
Flow diagram of study 2. Facial image reproduced with permission from Bai et al. (2005). Food image reproduced with permission from Kong (2012).

The experimental conditions are the same as those in study 1. Before the experiment began, the participants were asked to rate their current hunger level (0 being equal to not hungry and 10 being equal to very hungry). The experiment (see Figure 2) was then carried out. First, the participants were informed of the experimental tasks. They next entered the practice. After the practice, a formal investigation was carried out. In each trial, a gaze point “+” of 500 ms appears in the center of the screen, followed by a front masking stimulus of 100 ms, followed by a start-up picture of 17 ms, followed by a back masking stimulus of 100 ms, and then a food picture is presented. Participants need to rate their willingness to eat at 9 points, where “1” means they do not want to eat very much, and “9” means they want to eat very much. After pressing the key, the food screen disappears, and there is a time interval of 500 ms between the two trials. The experiment was divided into two parts. Positive and negative emotional priming were carried out separately. Each part had 2 (priming map) × 30 (food map) combinations, and each combination was repeated once with 120 trials. The two sections were separated by 15 min.

Interviews were conducted after the experiment. The interviews include two questions: (1) when completing the simulated eating task, did you see a face picture flashing quickly before and after the food picture? If so, please describe the image you saw; (2) please guess the purpose of the experiment.

### 3.2 Results

#### 3.2.1 Emotional priming effect test

Interviews were conducted after the experiment. Two participants indicated that they could see the light and shadow flashing before and after the food pictures, however, they could not see the specific content on the image clearly. One subject articulated that he could see two images of sad male faces flashing clearly, while the other participants were unaware of the subliminal stimulation. Therefore, the results indicated that the grading of food pictures is based on the participant’s willingness to eat and that the participants are not aware of the potency of the priming materials and the impact of priming stimulation. It is assessed that the experiment achieved the goal of subliminal priming.

#### 3.2.2 Eating willingness of successful/unsuccessful/unrestricted dieters in subliminal emotional priming state

In employing the back testing technique, it was determined that the willingness to eat when was is in a positive emotional state was significantly higher than when one is in negative states. In this context, ‘back testing’ refers to a method in which the data or calculations are reevaluated using an alternative approach to confirm initial results. The analysis results further revealed a significant main effect for emotional valence (*F* (1, 87) = 5.351, *p* < 0.05, *η*2 = 0.058). This reaffirms that subliminal stimulation effectively altered the emotional state of the participants, demonstrating the efficacy of subliminal emotional induction. The interaction, however, was not statistically significant (*F* (1, 87) = 1.561, *p* = 0.216, *η*2 = 0.035), as shown in Table 4.

**Table 4:** Eating willingness of three groups of participants in positive and negative subliminal emotional priming state (M ± SD).

	Unrestricted dieters (*n* = 30)	Successful dieters (*n* = 30)	Unsuccessful dieters (*n* = 30)
Positive	4.41 ± 1.31	4.65 ± 1.07	4.82 ± 1.08
Negativity	4.40 ± 1.31	4.43 ± 1.38	4.41 ± 1.27

## 4 Discussion

### 4.1 The influence of emotions on the eating willingness of restrictive dieters

Based on the results of the two studies, positive emotional stimulation increases the appetite of restrictive dieters at both conscious and unconscious levels. The appetite of unsuccessful dieters is higher than that of successful dieters and unrestricted dieters. In terms of craving for food, the unsuccessful dieters exhibit a stronger desire than the other two types of dieters. The results of this study are inconsistent with the previous research results that restrictive dieters have more eating willingness under negative emotional conditions. There are many theories through which to explain the phenomenon of restrictive dieters increasing their food intake in a negative emotional state, such as the Escape Theory (Heatherton and Baumeister, 1991), the Masking Theory (Chao et al., 2016) and the Emotional Adjustment Model (Adam and Epel, 2007; Stephanie and Finlayson, 2011), Limited Resource Hypothesis (Boon et al., 1998). Among them, the Hypothesis of Limited Resources can also explain the phenomenon of increased eating under positive emotions. This hypothesis holds that cognitive resources are limited, and positive emotions also occupy a part of cognitive resources in their minds. The cognitive resources used by restrictive dieters for dieting goals are occupied by coping with positive emotions, which also leads to increased eating.

Moreover, the enhancement of eating willingness in positive emotions stems from the influence of happiness on cognitive and motivational processes and the enhancement of the ability to deal with stimuli. Sadness is related to cognitive processes and a reduction in sporting activities, while negative emotions have been associated with a reduced motivation to eat and finding pleasure in food. In positive emotions, dieters enlarge their feelings about food, improve their evaluation of food, and increase their motivation to eat in order to enjoy food. In negative emotions, the hedonic response to food decreases. Moreover, Macht (2008) found that happy people have a higher evaluation of taste pleasure and food stimulation effect than sad people, showing a tendency to eat more food. Additionally, from the perspective of evolution, negative emotion is a response to potentially life-threatening information inherited by human beings for better survival and firmly engraved in the collective subconscious. Compared with negative emotions, positive emotions cannot solve survival-related problems but can make individuals live better (Fredrickson and Barbara, 1998). The induction of emotions can relieve the eating inhibition of restrictive dieters. However, when negative emotions are induced, they can also make the restrictive dieters more alert to the stimulation leading them to exercise restraint in eating. Compared with negative emotions, positive emotions make people happy, and it is easy to eat more inadvertently in a relaxed state. This pattern closely mirrors people’s life experiences. Usually, people celebrate by eating when they are happy, and when they are in a good mood, they will feel their appetite is wide open, and their appetite is greatly boosted.

In addition, the experimental materials and methods will also affect the experimental results. In this study, sad and happy face pictures were used to induce negative and positive emotions, while Cools et al. (1992) used horror and comedy movies to influence negative and positive emotions. On the one hand, there are differences in the emotional intensity generated by the two materials, and different emotions also impacted food intake (Macht, 2008). On the other hand, happiness, anger, sadness and fear have different functions. As an example, anger has a high degree of arousal and mobilizes energy to the greatest extent; Fear makes people escape from danger; Sadness reduces people’s activity; Happiness stimulates individual kinetic energy. Sad faces, in comparison to angry and scared faces, produce a lesser degree of threat and have a weaker early warning effect, leading to a diminished priming effect In this study, the priming effect caused by the subliminal faces showing happiness and sadness reached a significant level, and the participants had a higher willingness to eat under the priming of positive emotions, which affirmed the successful implementation of the subliminal experiment and this unconscious emotion influenced the performance of subsequent tasks. Using the subliminal emotional priming paradigm to study restrictive dieters is the essential exploration of the emotional eating of restrictive dieters.

### 4.2 The difference of eating willingness between successful and unsuccessful dieters and unrestricted dieters

In a positive mood, the eating willingness of unsuccessful dieters is higher than that of successful dieters. One explanation being that the cognitive resources of the unsuccessful dieters are subordinate to those of the more successful dieters, and when the cognitive resources are occupied by emotions, they cannot spare other cognitive resources to monitor eating. Another explanation is that unsuccessful and successful dieters have different dieting goals. The unsuccessful dieters set a lower threshold of dieting boundary. When they eat the same amount of food, they may have reached the dieting limits of successful dieters yet not the threshold of unsuccessful dieters, so they have a stronger inclination to eat. Another explanation is that restricted dieters have hedonic and dieting goals, and unsuccessful dieters beat dieting goals under the influence of emotions, thus increasing their eating behavior. However, successful dieters can maintain dieting goals because of their successful dieting experience, although they have activated hedonic goals because they have robust self-regulation mechanisms (Wolfgang et al., 2013).

The subliminal emotional priming experiment in study 2 is not significant in interaction. Although the main effect is significant, which shows that the participants have a higher willingness to eat with positive emotions, it is likely caused by restrictive dieters, and the appetite of unrestricted dieters has almost no change in positive and negative emotions, and the difference is not significant. The results of the first study show that the difference in eating willingness of unrestricted dieters with different emotions is the smallest, which is smaller than that of the successful group and the unsuccessful group. Based on the results of the two studies, it can be inferred that unrestricted dieters are less affected by emotions when eating. The possible reason is that the emotional processing ability of unrestricted dieters is weak, or their emotional sensitivity is low.

Additionally, unrestricted dieters may be at reduced vulnerability to emotions, and there may also be different priming stimuli from those of restrictive dieters. Originally, in subliminal experiments, priming stimuli left only a shallow impression or even no impression on attention, and the emotional susceptibility of unrestricted dieters was low. Moreover, the appetite for eating under the two kinds of emotions was equal, and they were assessed more according to the target stimulus attributes. In addition, it may also be related to the allocation of psychological resources. Subliminal emotional priming is a conscious process with sufficient psychological resources, while subliminal emotional priming is an unconscious process with insufficient psychological resources. The distribution of psychological resources leads to the difference between the two studies. Resources are limited. Unrestricted dieters have no goal of shaping their bodies. Compared with restrictive dieters, they have more resources, are relatively abundant in coping with emotions, and will have no fluctuations in eating.

## 5 Conclusion and prospect

To sum up, this study found that positive emotion is an essential factor affecting the increase of food consumption of restrictive dieters, especially for those who fail to restrict food. Under the priming of subliminal and subliminal emotions, the main effects of emotional valence are significant and consistent. The above results show that positive emotional stimulation will increase the appetite of restrictive dieters at both conscious and unconscious levels. Moreover, the most significant contributor to this increase is the unsuccessful dieters. It can be seen that the craving for food is increased in the unsuccessful dieters than for the other two types of dieters. Emotion can greatly disrupt their dieting boundaries, often leading to their dieting failure.

In this study, the stimuli used to elicit emotional responses (referred to as ‘expression materials’) are singular and lack of ecological validity. In future research, we can employ a wider variety of these emotional stimuli to explore the influence of more types of emotions on the eating behavior of restrictive dieters. The judgment of participants’ eating behavior is based on their response to two-dimensional picture stimuli, however, the taste and shape of food will also affect their eating behavior. In the future, we can adopt more realistic stimuli, such as using real food to investigate food intake; In addition, with people’s general concern about obesity and health problems, we can also examine the characteristics of restrictive eating behaviors of various groups, such as men and the elderly.

## Data availability statement

The data analyzed in this study is subject to the following licenses/restrictions: the raw data supporting the conclusions of this article will be made available by the corresponding authors, without undue reservation. Requests to access these datasets should be directed to WX, xinyun0501@163.com.

## Ethics statement

The studies involving humans were approved by School of Education Science, Chongqing Normal University. The studies were conducted in accordance with the local legislation and institutional requirements. The participants provided their written informed consent to participate in this study.

## Author contributions

YL: Writing – review & editing, Writing – original draft, Supervision, Methodology, Formal analysis, Conceptualization. YC: Writing – original draft, Methodology, Investigation, Data curation, Conceptualization. WX: Writing – review & editing, Project administration, Data curation, Conceptualization.
